# Community Living, Intellectual Disability and Extensive Support Needs: A Rights-Based Approach to Assessment and Intervention

**DOI:** 10.3390/ijerph18063175

**Published:** 2021-03-19

**Authors:** Laura Esteban, Patricia Navas, Miguel Ángel Verdugo, Víctor B. Arias

**Affiliations:** Institute on Community Inclusion and Department of Personality, Assessment and Psychological Treatments, University of Salamanca, 37005 Salamanca, Spain; lauraestesa@usal.es (L.E.); verdugo@usal.es (M.Á.V.); vbarias@usal.es (V.B.A.)

**Keywords:** intellectual disability, extensive support needs, rights, community living, privacy, rehabilitation, professional practices

## Abstract

People with intellectual disability (ID) and extensive support needs experience poorer quality of life than their peers whose disability is not as severe. Many of them live in residential settings that limit community participation and prevent them from exercising control over their lives. This work analyzes the extent to which professional practices are aimed at promoting the right to community living for people with ID and extensive support needs, as well as the rights that are particularly linked to it, such as the right to habilitation and rehabilitation and the right to privacy. A specific questionnaire was designed and administered to 729 adults with intellectual disability (*M* = 37.05; *DT* = 12.79) living in different settings (family home, residential facilities and group homes). Measurement and structural models were estimated using exploratory structural equation modeling. Results obtained reveal that people with extensive support needs receive less support in terms of guaranteeing their right to independent living and privacy, especially when they live in disability-related services. This study highlights the need to implement and monitor, using valid and reliable indicators, mesosystem strategies that guarantee the right to live and participate in the community, especially for individuals with ID and extensive support needs.

## 1. Introduction

The United Nations Convention on the Rights of Persons with Disabilities is based on the principles of non-discrimination, equal opportunities and accessibility [1], and it is aimed at promoting, protecting and ensuring the full and equal enjoyment of all human rights and fundamental freedoms by all persons with disabilities (p. 4) by means of appropriate modifications and adjustments. However, as some authors point out [2], changes in paradigms and models of care guided by the aforementioned principles have been almost exclusively limited to individuals with intellectual or developmental disabilities with higher levels of functioning. Thus, people with intellectual disabilities and extensive or generalized support needs are more likely to be placed in segregated institutions (settings in which, regardless of size, individuals do not have the right to exercise control over their lives). In these contexts, professional practices offer few opportunities for self-determination and participation, under the assumption that the inclusion of people with greater support needs is difficult to achieve [3]. These beliefs undoubtedly impact the work performed by professionals, providing fewer opportunities for participation [4,5] and potentially calling into question inclusion as a long-term goal within person-centered plans [6]. This situation results in a lack of compliance with the rights set forth in the Convention [1] and, in particular, with the right to live independently and participate in the community (Article 19). For these reasons, this study aims to analyze the extent to which professional practices promote the right of participating and living in the community of people with intellectual disability and extensive support needs and the extent to which the living environment and the support provided in it promote greater or lesser compliance with the Convention.

Before going into detail about the situations of exclusion still faced by this group, especially with regard to their inclusion and participation in the community, it is necessary to offer a definition of what the literature understands by people with intellectual disabilities and extensive support needs, given that the imprecise conceptualization of this population group consequently affects the way in which assessment protocols and plans for the provision of services and supports are operationalized [7].

People with intellectual disability and extensive support needs constitute a very heterogeneous group [7,8]. This group, which has also been described as people with profound intellectual and multiple disabilities, is generally characterized by severe limitations in intellectual functioning and adaptive behavior. Likewise, people in this group might also experience motor disorders, sensory deficits, severe communication problems and other physical or mental health conditions [9,10,11,12,13]. This group also includes people who do not share the mentioned characteristics but present severe challenging behaviors that significantly limit their functioning to the extent that they require extensive or generalized support in their daily life [13].

Although these characteristics are frequently mentioned to define this group of the population with intellectual disability (ID), the context (understood not only as the physical environment surrounding the person, but also as their social network and systems of support) plays an important role in the configuration of a person’s support needs [1,14]. Intellectual disability should be, therefore, understood as a state of functioning [15] that results when an individual (with his or her limitations and strengths) interacts with his or her environment [1,14,15]. This state of functioning can be improved if the person is provided with appropriate supports [15], so some contexts will be more “facilitating” than others and will contribute more to reducing the intensity of an individual’s support needs [16]. However, people with more severe disabilities have been traditionally considered “incapable” of participating in decision-making about their own lives, regardless of the support they receive, especially when they lack verbal capacity or have communication difficulties [17,18,19]. This way of thinking has resulted in considering that that person’s needs and desires will remain unchanged throughout his or her life and can be satisfied with the same professional services and supports, not paying much attention to how the context and professional practices could be modified to reduce support needs.

Individuals with intellectual disabilities and extensive support needs can account for up to 0.4% of the general population [20]. In the specific case of Spain, where this research is framed, the figure stands at 0.16%, which means a total of 63,610 people [8], a number that is similar to the figures reported for countries such as Finland (0.13%) or the United Kingdom (0.2%) [21,22]. Despite this significant number, they are hardly taken into consideration in public policies and research [23]. This translates into a reduced number of studies on their quality of life [2,24], even though it is known to be poorer than that of people with lower support needs [25,26]. Indeed, having extensive support needs is the best predictor of the worst results in the different areas that account for good quality of life [27,28]. Similarly, other studies suggest that lower levels of adaptive behavior [29,30], greater severity of the intellectual disability (ID) [31] or the presence of challenging behaviors [32] are associated with poor personal results.

The worst personal results obtained by people with intellectual disability and extensive support needs are especially apparent in areas such as self-determination or social inclusion [27,33]. Likewise, and although research has repeatedly proven that living in community environments fosters this group’s self-determination [3,17,34], those whose disabilities are more severe are less likely to live and participate in ordinary settings [4,35], especially if they have exceptional medical or behavioral needs [36]. The limited experiences of community participation that are available to them due to their living in segregated contexts, place them at increased risk of social exclusion [6,33].

Despite the limited opportunities afforded to this group of individuals with ID, research so far has revealed that the transition into the community of people with extensive support needs is related to improvements in adaptive behavior [37,38], self-determination [39,40], interpersonal relationships [37], participation [39] and overall quality of life [40,41]. In fact, according to several studies [40,41,42], people with intellectual disability and extensive support needs benefited more from the transition to community than those with ID who required less support.

Notwithstanding the benefits to people with ID and extensive support needs of individualized support in community settings, this group is often excluded from these services because of the additional costs that could be involved [11]. This discrimination is against Article 19 of the International Convention on the Rights of Persons with Disabilities [1], which establishes that State Parties will provide the necessary support services (including personal assistance) so that all people with disabilities, regardless of type or severity, may enjoy a fully independent life (with the support they may require) in the community. Independent living does not mean living autonomously, without support or unmet needs [43]. Independent living should be understood as a life of choice, where we all need support in our daily lives and we also support others. This concept of interdependency should be emphasized because, as noted above, the term “independent” can deny people with more severe disabilities fundamental rights by thinking they will never reach such independence due to the intensity of their support needs, and blur society’s responsibility to personalize the supports that, in a “disabling” context, the person may need. People with extensive support needs can participate in their community when they have appropriate support [44].

In the case of Spain, as revealed in the last report on the process of deinstitutionalization in Europe, no specific policy to promote life in the community for people with disabilities has been developed [45], which particularly affects people with more severe intellectual and developmental disabilities. A study based on the most recent data obtained for this country [13] reveals that this social group still faces many situations of social exclusion and violation of rights. Thus, 16,591 people with extensive support needs continue to live in institutions and the support they receive is mainly focused on residential care (32.4%) and the assistance provided by day-care centers (28.2%), with other resources to promote inclusion in community living being vestigial [13]. These data reflect the institution-based model that prevails in Spain for those people with more significant disabilities, to the detriment of other services that are more inclusive and are consistent with the contents of Article 19 of the Convention, such as personal assistance [46,47]. This situation is a matter of concern to the Committee on the Rights of Persons with Disabilities, which, in its latest report on Spain, criticized the barriers to personal assistance services, as well as the constant investment of public funds in residential institutions [48].

The segregation of people with intellectual disability and extensive support needs not only violates their right to be provided with the necessary support to live in the community (Article 19 of the Convention), but it also affects others, such as the right to habilitation and rehabilitation (i.e., the right of people with disabilities to attain and maintain maximum independence and to full inclusion and participation in all aspects of life, which is gathered in Article 26 of the Convention) or the right to privacy (Article 22). These rights can be severely violated when people live in specific, non-natural contexts [49].

It is therefore essential to monitor the implementation of the Convention through the use of precise instruments, assessing how the support provided to people with ID and extensive support needs ensures compliance with the rights outlined in this international treaty [8,13,50]. In this regard, and in relation to the right to living independently and being included in the community that concerns this study (Article 19 of the Convention), it should be noted that living in community settings is a necessary but not sufficient condition to achieve the goal of inclusion of people with ID [45]. That the person can live and participate in the community involves a major change in the provision of support, requiring person-centered professional practices [33,51,52,53,54,55]. Hence, despite living in community settings where there are enough professionals available, many people with significant disabilities may spend a large part of their time alone, without participating in activities that they are interested in [4,5,56].

The aim of this study is, therefore, to analyze the extent to which professional support is aimed at promoting the right to community living for people with intellectual disability and extensive support needs, as well as the rights that are particularly linked to it, such as the right to habilitation and rehabilitation and the right to privacy, as specified in the following aspects:

(a) Analysis of the extent to which a set of indicators contributes to measure the latent variables under study (i.e., professional practices aimed at promoting the right to living independently, alongside those of habilitation and rehabilitation and privacy of people with ID).
(b) Analysis of the differences in these latent variables between people with ID and extensive support needs and people with ID and less intense support needs.
(c) Analysis of the interaction effect between the home environment where people with ID live and the differences among the latent variables under study. (i.e., professional practices aimed at promoting the right to living independently, habilitation and rehabilitation and privacy).

## 2. Materials and Methods

### 2.1. Participants

The total sample included 729 adults diagnosed with ID. This sample was divided into two subsamples according to the level of the person’s support needs: high support needs (i.e., extensive support needs) (n = 470) and low support needs (n = 259). Table 1 shows the demographic characteristics of all the participants grouped according to their support needs.

The distribution of men and women in both groups of people with ID was equiprobable (χ^2^ (1, N = 729) = 0.47, *p* = 0.49). There were no statistically significant differences between groups in regards to age. Although the need for more extensive support to perform everyday activities can be the result of other reasons beyond the severity of the ID, there was a statistically significant association between this variable and the intensity of the support required (χ^2^ (2, N = 729) = 252.05, *p* < 0.001). Thus, while only 9.7% (N = 25) of those with low support needs had been diagnosed with severe or profound ID, such percentage rose to 68.9% (N = 324) among individuals with extensive support needs. Moreover, the latter had twice the risk of suffering from an associated disability (*p* < 0.001, CI 95% [1.7, 3.2]), this being a physical or sensory disability in most cases (39.5% and 26.4%, respectively).

All the information regarding the sample was obtained through 429 informants, all of them professionals belonging to different organizations that provided support for people with ID. Most of them were women (78.8%), with an average age of 39.86 (*SD* = 9.13). The average time of contact with the person with ID was 7.26 years (*SD* = 6.1) the frequency of contact in most cases being daily (82.7%).

### 2.2. Instrument

The data used for the purposes of this study belong to a broader line of research whose aim was to assess the extent to which disability organizations engage in actions aimed at defending and guaranteeing the rights of people with ID that are defined in the Convention [1]. With this purpose, after a review of the scientific literature [8] and a thorough analysis of the Articles stated in the Convention, a 52-indicator questionnaire was developed. This initial set of indicators was shared with a team composed of eight experts, who were relatives of people with ID (n = 3) and professionals (n = 5) belonging to disability organizations. After this analysis, 19 new indicators were added, so that the final set encompasses 71 indicators related to the Articles of the Convention.

The questionnaire, which is to be completed by a professional who knows the person with ID whose rights are being assessed very well, is aimed at measuring how the organization that provides support engages in the practices addressed in each item, based on the following response format: 1 = *Never* (i.e., the statement completely fails to reflect what is being done by the organization that the person with ID attends), 2 = *Sometimes* (i.e., the statement reflects an action that is from time to time or sporadically performed by the organization), 3 = *Frequently* (i.e., the statement reflects an action that is frequently, but not systematically, performed by the organization), 4 = *Always* (i.e., the statement perfectly reflects what is being done by the organization that the person with ID attends).

Since this study analyzes the extent to which professional support is aimed at promoting the right to living in the community of people with ID and extensive support needs (Article 19 of the Convention), as well as the aspects that are particularly linked to it, such as the right to habilitation and rehabilitation (Article 26) and the right to privacy (Article 22), Table 2 includes the items developed. Should the reader be interested, a copy of the entire questionnaire may be requested from the corresponding author.

### 2.3. Procedure

The research team initially contacted a total of 261 disability organizations that are part of the main provider of support for people with ID in Spain, *Plena inclusión*, which is made up of 900 local entities. These organizations were contacted in view of their previously expressed interest in collaborating in a broader study on the rights of the people with extensive support needs in Spain [8]. Finally, 77 organizations took part in the study (29.5%). Since the purpose of this research is to explore the extent to which professional support contributes to guaranteeing the rights of all the people with ID, regardless of the support needed, we asked each of the participating organizations to randomly select a number of users with extensive and low support needs within their organization in order to explore possible differences in the provision of professional services to these two groups of ID population. Criteria for this selection were based on the size and characteristics of each organization.

To favor the identification of users with extensive or low support needs, the organizations were given an operational definition of people with extensive and generalized support needs, whose content appears in the introduction to this article. The score achieved by the people with ID assessed using the Supports Intensity Scale (SIS) was also requested [57], confirming that 99.0% of the people identified by the professionals as “people with extensive support needs” obtained a SIS score that was consistent with the need for extensive or generalized support needs.

The professionals who completed the questionnaire were also given a summary of the Articles gathered in the Convention, the questionnaire described in the previous section, an instruction sheet and the informed consent to be filled out by the professional and the person with ID to be assessed (or his/her legal guardian where appropriate). All this was accompanied by a letter that included the specifics of the research and the contact details of the research team to solve any query. All the procedures performed in this research were in accordance with the 1964 Declaration of Helsinki and its subsequent amendments. This procedure was also approved by the Bioethics Committee of the University of Salamanca (registration number 434).

### 2.4. Data Analysis

Measurement and structural models were estimated using exploratory structural equation modeling, ESEM [58,59]. ESEM integrates the procedures of exploratory factor analysis (EFA) and confirmatory factor analysis (CFA). Thus, ESEM combines the flexibility of EFA with the main advantages of CFA, including estimation of standard comparative fit indices across nested models, the possibility of evaluating and modeling sources of local misfit, or the capacity to perform invariance and multi-group analysis, generally more efficiently and realistically than specifications based on the independent-cluster model of confirmatory factor analysis [60]. ESEM models were estimated using oblique target rotation. Target rotation finds the rotated solution that is closest to a pre-specified loading pattern, enabling ESEM to be used in a semi-confirmatory mode [58].

The analysis was performed in three stages. The first involved examining the extent to which the empirical structure of the data was coherent with the theoretical structure using an ESEM factor model to evaluate goodness-of-fit. After defining the measurement model, the testing for metric invariance among groups included as predictor variables in the SEM model (i.e., sex, age, support needs, disability, comorbidity and living setting) was performed. This was done using ESEM-MIMIC models (multiple indicators-multiple causes) as recommended by Morin, Arens and Marsh [61]. According to this procedure, three MIMIC models were compared for each grouping variable: a saturated model that hypothesizes scalar noninvariance, an invariance model that hypothesizes full scalar invariance and a null model that hypothesizes the lack of relationship between grouping variables and latent variables. If the fit of the saturated model is better than that of the invariance model, it is advisable to examine whether or not the problem is generalized to all regression parameters, in which case the feasibility of goodness-of-fit of a partial invariance model according to the impact of noninvariance on the estimator of latent means is estimated. The third stage involved the estimation of the final SEM model and the comparison of its fit with that of alternative specifications.

All models were estimated from polychoric correlation matrices using weighted least squares mean and variance (WLSMV), adjusted for estimation, given the ordinal nature of the input data [62]. Goodness-of-fit was evaluated using the comparative fit index (CFI), the Tucker-Lewis index (TLI), the root mean square error of approximation (RMSEA), the modification indices (MI) and the standardized expected parameters of change (SEPC). For the CFI and TLI indices, estimated values above 0.90 and 0.95 indicate acceptable and good fit, respectively [63]. For the RMSEA, values equal to or lower than 0.05 and 0.08 are considered good and acceptable, respectively [63,64]. MI greater than 10 together with SEPC greater than 0.20 suggest the presence of local misfit that should be investigated [65,66,67]. All analyses were performed using MPlus v.7.3 (Muthén & Muthén, Los Angeles, CA, USA) [68].

## 3. Results

### 3.1. Analysis Using ESEM Models of the Extent to Which the Set of Indicators Developed Allows the Study, Minimizing Measurement Error, of the Latent Variables That Are the Object of Research: Right to Living Independently and Being Included in the Community, Right to Privacy and Right to Habilitation and Rehabilitation

The fit of the ESEM three-factor model was acceptable (RMSEA = 0.075, CFI = 0.985; TLI = 0.968). After inspecting possible sources of local misfit, seven correlated residuals with modification indices and SEPC values above 10 and 0.3, respectively, were observed. To begin with, the parameter with the largest misspecification was freed (correlation among residuals of items C3 and C4; MI = 44, SEPC = 1.4). The modified model achieved substantially better fit (RMSEA = 0.055; CFI = 0.992; TLI = 0.982), with no more free parameters with SEPC > 0.3. The initial standardized loadings of the modified model were reasonably large (.44 to 0.94, mean = 0.70) and consistent with the scale’s theoretical structure (see Table 3). Cross-loadings were generally small (0.001 to 0.28, mean = 0.09) and explained common variance in each item according to the initial loading which was between 73% and 99%, suggesting that each item has adequate discrimination power in its corresponding theoretical factor.

Table 4 shows the fit indexes for the MIMIC models estimated for each grouping variable. The results in Table 4 can be interpreted as follows: for each grouping variable (i.e., sex, age, etc.) the fit of three MIMIC models is compared. If the best fit is observed in the null model, then (a) there is no relevant measurement bias contaminating comparisons between groups and (b) there are no differences between groups in the measured variables (e.g., there are no substantial differences between people according to sex, age, etc. in those variables under study: independent living and community participation, privacy and habilitation and rehabilitation). If the best fit is shown by the invariant model, then (a) there is no measurement bias and (b) there are substantial differences between groups in the measured variables. Finally, if the best fit is shown by the saturated model, then (a) there is probably some degree of measurement bias, which (b) makes meaningful comparisons between groups unfeasible, unless measurement bias is concentrated in a limited number of items (which makes it possible to estimate a partially invariant model, controlling the bias in the affected items).

The null model showed best fit for sex, age, degree of disability and presence of other disabilities. This means that (a) model invariance was sufficient for group comparisons to be meaningful, but (b) group differences were irrelevant. According to the parameters of the invariant models, no differences in groups (sex and presence of another disability) or correlation (age and degree of disability) were significantly different from zero (*p* < 0.05) in any of the three factors.

In regards to support needs, the saturated model showed slightly better fit than the invariant model (suggesting lack of invariance) and the null model achieved poorer fit than its competitors (suggesting the presence of significant group differences). After inspecting the modification parameters, a new partially invariant model was estimated, freeing the intercept of item C3. The fit of this model was slightly better than that of the saturated model, suggesting that measurement bias was lower and limited to a single item. The differences in latent means estimated by the invariant and partially invariant models were compared, obtaining a negligible difference (0.02 standard deviation). This result suggests that the effect of the item’s differential functioning in the general model was irrelevant. Finally, in the case of the “living setting” variable, the invariant model obtained better fit than its competitors, showing sufficient invariance and the presence of significant effects of the covariate on the factors.

To verify the results described above and obtain the final estimations, three new models were estimated (Table 5). The exhaustive model included all the covariates simultaneously. The parsimonious model included all the covariates but setting the regression paths of the covariates that obtained a null effect in previous models to zero (this means simultaneous verification of the hypotheses that the variables sex, age, disability and comorbidity have no effect on respect for the person with disability’s rights). The interaction model included a new covariate based on the interaction between the support needs and type of residence variables.

The parsimonious model achieved slightly better fit than the exhaustive model in certain indices (ΔCFI = 0.001, ΔTLI = 0.001) and slightly worse in others (Δ χ2 = 20, ΔRMSEA = 0.001). Since misfit was minimal and located in the indices that were most affected by differences in parsimony, we decided to keep the parsimonious model. In regards to the interaction model, it achieved better fit than the parsimonious model in all indices (ΔRMSEA = −0.007, ΔCFI = 0.002, ΔTLI = 0.003) except for chi-square (Δ χ2 = 3). Although both models achieved good absolute fit, the presence of differences in most fit indices makes it advisable to conserve the interaction model and inspect its parameters before deciding on its retention.

### 3.2. Analysis of Differences in the Latent Variables under Study between People with Intellectual Disability and Extensive Support Needs and People with Low Support Needs

**Figure 1:**
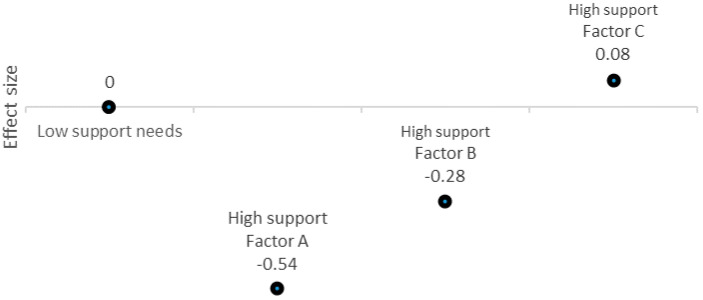
Mean differences according to the extent of support needs. Note: A, B and C factors correspond to professional practices oriented to promote the rights included in Articles 19, 22 and 26 of the Convention, respectively.

Figure 1 shows differences in latent means on each factor according to the extent of support needs (interaction model), expressed as effect size in relation to a mean setting at zero for the group of people with low support needs. Thus, each value represents the size effect of the difference between the group with low support needs (whose mean is always zero) and the group with extensive support needs on each of the examined factors: factor A (professional practices aimed at promoting independent living and inclusion in the community), factor B (professional practices that guarantee privacy), factor C (professional support that promotes habilitation and rehabilitation). The extensive support needs group achieved moderately lower scores on factor A (*p* < 0.01, *d* = 0.54), slightly lower on factor B (*p* < 0.01, *d* = 0.28) and no significant difference on factor C (*p* > 0.05, *d* = 0.08). Considering that lower scores on factors A, B and C indicate lower compliance with the rights set forth in the Convention, these results suggest, without taking into account any other variables that will be studied later, that people with extensive support needs experience more difficulties in receiving professional supports that promote their right to community living and privacy.

### 3.3. Analysis of the Interaction Effect between Living Environment and Differences in the Latent Variables under Study

**Figure 2:**
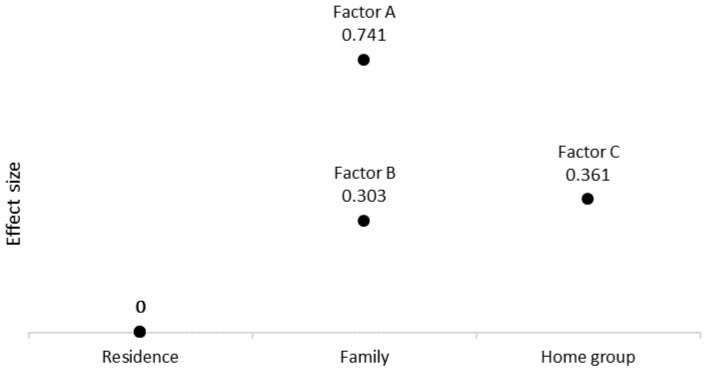
Size of mean differences by living setting (differences not significantly different from zero are not represented). Note: A, B and C factors correspond to professional practices oriented to promote the rights included in Articles 19, 22 and 26 of the Convention, respectively.

Figure 2 shows the differences in means on each factor according to the person’s living setting: residential facility (nursing home), group home (also known as supervised apartment in some countries) or family home (for reasons of clarity, the graph only shows differences that are significantly different from zero). People living at nursing homes were used as baseline level for the rest of groups (mean zero). On factor A (i.e., living independently and being included in the community), the group living with their family achieved substantially higher scores than the nursing home group (*d* = 0.74) and slightly higher than those living in a group home (*d* = 0.37) (the differences between family and group home were calculated by subtracting their effect sizes.). On factor B (i.e., privacy), individuals living with their families obtained slightly higher scores than the nursing home group (*d* = 0.30) and there were non-significant differences compared to individuals living in a group home or supervised apartment (*d* = 0.17, ns). Finally, those living with their family showed no significant differences on factor C (i.e., habilitation and rehabilitation) compared to any of the other groups (*d* = 0.07 and 0.02). The group of people with ID living in a group home scored slightly higher on factor C than the nursing home and family groups (*d* = 0.36). This difference, however, and as explained later in this section, is reduced once segregation according to level of support needs is included in the model.

**Figure 3:**
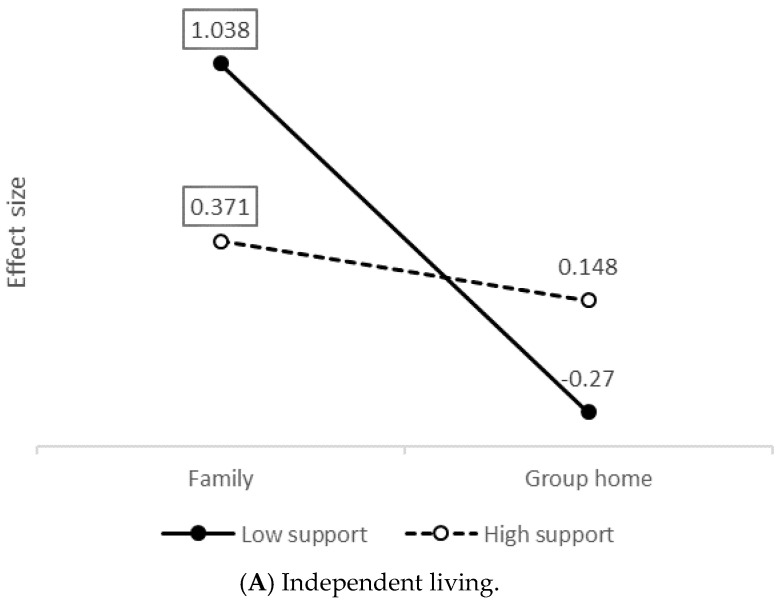

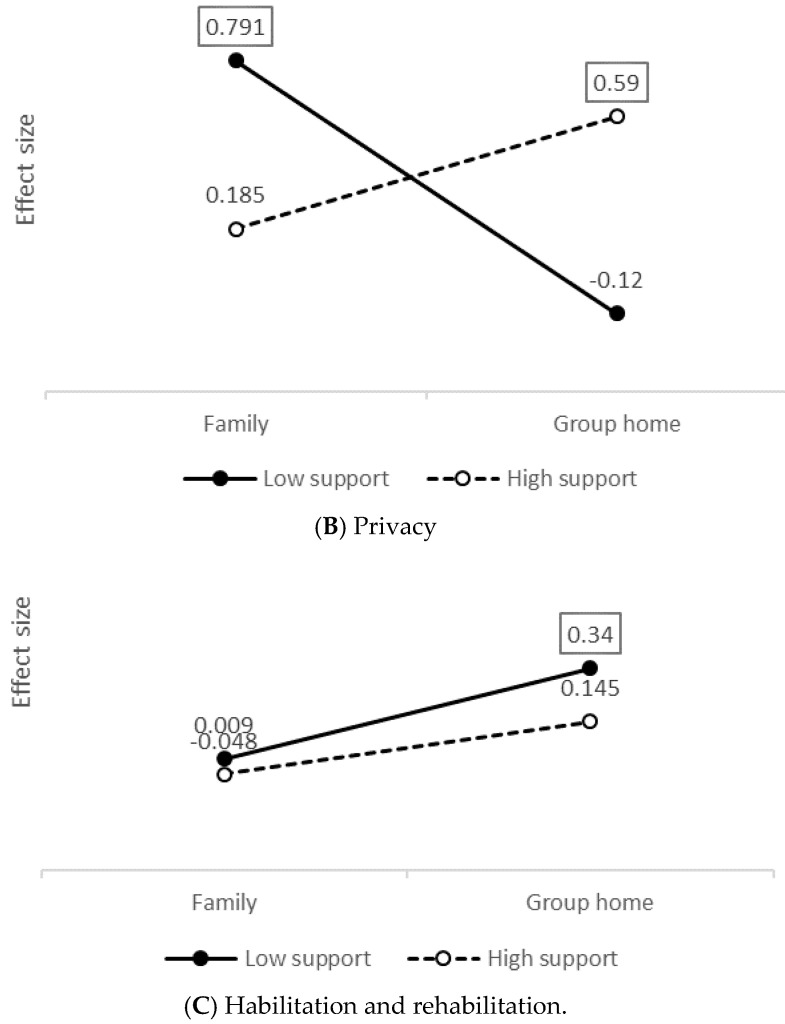
Size of mean differences according to degree of support needed and living environment. Note: (**A**–**C**) factors correspond to professional practices oriented to promote the rights included in Articles 19, 22 and 26 of the Convention, respectively. The rectangle indicates differences significantly different from zero.

Figure 3A–C shows the differences in latent mean between living environments, separately for extensive and low support needs. Each point on the graph represents the standardized mean difference (analogous to Cohen’s *d*) between those living in a nursing home (group that is being used as baseline level and its mean is set to zero) and the other two groups (family and group home) on each variable of interest, taking also into account the degree of support required (high support needs or low support needs). Differences that are significantly different from zero (*p* > 0.05) are provided inside a rectangle.

On factor A (i.e., independent living) there was a clear interaction effect: people living at the family home with low support needs obtained scores that were much higher than those of their nursing home counterparts (*d* = 1.08), while the same difference was much lower in the case of people with extensive support needs (*d* = 0.37). There were no significant differences (*p* > 0.05) between low and high support needs depending on whether they lived in a nursing home or a group home. Overall, we might conclude that, among people with extensive support needs, professional practices aimed at promoting the right to living independently (i.e., factor A) are similar across the three living settings, with a slight difference in favor of those who live with their family. On the other hand, in the group with low support needs, the family home seems to be by far the setting where the right to living independently is better satisfied by the professional support provided, with no noticeable differences between the nursing home and group home settings. 

On factor B there was once again a significant interaction effect. Among individuals with low support needs, guarantee of the persons’ right to privacy is greater when they live with their family than when they do so in a nursing home (*d* = 0.791) or a group home (*d* = 0.911), while it was similar between nursing home and group home (*d* = −0.12, ns). This effect varied inversely among people with extensive support needs: privacy was respected to the same extent in nursing home and family settings (*d* = 0.18, ns), but significantly more in group homes than in residential facilities or nursing homes (*d* = 0.59) and family settings (*d* = 0.40). This result indicates that the difference observed between nursing home and family across the entire sample (*d* = 0.30 in Figure 2) is not a generalizable effect, but a mixture between a significant effect (*d* = 0.79) and a non-significant effect (*d* = 0.18). Likewise, the lack of differences between group home and nursing home across the entire sample is not such, but the results of the mixture of a moderate effect (*d* = 0.59) and a non-significant effect (*d* = −0.12).

Regarding factor C (i.e., right to habilitation and rehabilitation) there were no significant differences in professional practices between living environments, against what had been observed before including segregation according to level of support needs. Consequently, the loss of statistical significance could be due to a reduction in the power of the instrument because of the smaller number of subjects in each group and the small magnitude of group differences. In terms of size effect only, a very weak interaction was observed, with no differences across groups except in the case of people with low support needs living in group homes, whose scores on the “habilitation/rehabilitation” factor were slightly higher than those obtained for nursing home and family (*d* = 0.34). We can therefore conclude that there are possibly no substantial differences in living settings regarding the degree of implementation of measures aimed at guaranteeing the right to habilitation/rehabilitation, regardless of the extent of the person’s support needs. 

## 4. Discussion

The main goal of this study was to analyze the extent to which professional practices are aimed at promoting the right of participating and living in the community of people with intellectual disability and extensive support needs, as well as their right to privacy and to habilitation and rehabilitation, both of which are aspects related to the former [49].

According to the results, it can be affirmed that organizational and professional practices aimed at guaranteeing the right to living independently are more frequent when the needs of the person with ID are not so extensive. This is consistent with the findings of previous studies that suggest that people with extensive support needs receive less support aimed at their social inclusion [69] or that the funding of the support they need to live independently is not enough [70]. Although not as noticeable, this is also the case with supports aimed at ensuring their privacy. As already stated by Björnsdóttir and Stefánsdóttir [71], people who require extensive support suffer from a lack of privacy that also threatens other rights such as the right to sexuality. Thirdly, the results of this study suggest that all people with ID, regardless of their support needs, benefit from professional practices aimed at fostering their right to habilitation and rehabilitation. The advanced implementation of person-centered planning in Spain [72,73] could explain the lack of differences in the assessed indicators (aimed at verifying whether work with the person is done based on their likes and preferences and in community settings) according to the person’s support needs. 

Another of the objectives was to analyze the extent to which the living environment and the support provided in it promote greater or lesser compliance with the assessed rights. Regarding the right to living independently and being included in the community (Article 19 of the UN Convention), the results show that those who live with their family receive professional support that is more oriented towards ensuring the fulfillment of this right, especially if they have lower support requirements. The existence of a support network for those who live with their families could contribute to explain these results, since natural, alongside professional support, plays an important role in the fostering of independent living [74]. Given the role that families play in promoting independent living [75,76], any process aimed at fostering the participation of the person with ID in his or her community should focus not only on the person, but also on the family unit, making it part of the process as the family members themselves claim [77]. It is therefore important to attend to their needs and remember that they may also require support to adjust to possible changes in the lives of their relatives with ID [77]. Both person-centered planning and family-centered planning should be combined, preparing the family for changes and addressing their needs [78]. When families are involved, initial doubts and fears shift, over time, to satisfaction with the life their family members with ID have in their community [77,79]. In these processes of person- and family-centered planning, public policies should promote the funding of supports such as personal assistance and community resources, so that the family does not experience an overload of care that may interfere with its role as a catalyst for the inclusion of his or her family member [13].

It is surprising to find out that there are almost no differences in the participation and living in the community indicators between those who are living at a residential facility and those who live in a supervised apartment (group home) in their community, regardless of the person’s support needs. The reason for this could be that independent living services such as group homes often differ from nursing homes in size or location, but still keep some of facilities’ characteristics [54], such as rigid routines, social distance or depersonalization [80]. As noted by certain authors [81,82] the type of home might be not as important as the type of support provided, which should be focused on the person and encourage participation. It is essential for organizations that provide support for people with ID to adopt a Quality of Life Supports Model, focused on personal results, to replace traditional care models [83,84,85].

Regarding the right to privacy (Article 22 of the UN Convention), the study’s results show that when the person’s support needs are lower and they live with their family, the organization makes more efforts to guarantee their right to intimacy. These results are consistent with those obtained in previous studies where family is suggested to provide crucial support, although it can also become a source of important obstacles if, despite professional support, it overprotects and exerts control over the person with disability [75,86], this overprotection being greater towards people with extensive support needs [17,19] according to the data presented. Once again, the fact that professional practices are less oriented towards guaranteeing respect to privacy when the person lives in an institutional setting, reveals the need to revise our support provision model in organization-linked contexts [48].

Finally, professional practices are always geared towards seeking maximum independence for the person (Article 26 of the Convention), regardless of the person’s living environment or support needs. These results are consistent with the observations gathered in the latest report of the Convention’s committee in Spain [48], where no violation of the right to habilitation and rehabilitation is detected.

Nevertheless, the results of this study should be taken with caution, since it is not free from limitations. The most important of these is the fact that the items included and analyzed are focused on the actions engaged in by the organizations rather than on those performed by the people within the person with ID’s closest social circle. Therefore, it would be advisable to examine family practices and how they align with the articles of the Convention, since, according to Björnsdóttir et al. [17], the support provided in the family home may have characteristics that are typical of that provided by institutions.

Another of the limitations of this study is that variables related to the organization and its culture could not be controlled. Results might vary if factors such as the number of people allocated to each professional, leadership or training of the professionals, or engagement of the organization with the inclusion of people with ID in community settings were to be considered, since all these aspects have an impact on the provision of support that may favor the person’s participation and inclusion [87]. Examining such factors and their relation to the results is important, since, unlike the personal variables addressed, they are also potentially modifiable.

Although the study sample is large (N = 729), the total number of people living in group homes or supervised apartments is another limitation due to its size (n = 34). It would be advisable to rely on a larger sample size in this group, which would mean an improvement in the power of the analysis, allowing the identification of small but substantively relevant effects.

The relevance of this work lies in the importance of providing data on the measures taken by organizations to promote the exercise of rights of a traditionally neglected group such as people with intellectual disability and extensive support needs. Among the analyzed rights, emphasis should be placed on the importance of living independently and being included in the community, both because of its cross-cutting nature and because it is essential for full enjoyment of the rest of the rights laid down in the Convention [88]. Moreover, this right is especially linked to a central area of people’s lives as is self-determination, where people with extensive support needs experience lower levels of satisfaction than those with less severe disabilities [28,29,30].

The results presented here show that deinstitutionalization remains a challenge for the European Union, as noted in the latest report on transition to community-based services in 27 EU member states [41]. This report concludes that “the number of people in institutions does not appear to have changed substantially over the past 10 years” (p. 3), with people with ID and more extensive support needs most likely to continue living in institutional settings. The Academic Network of European Disability experts highlights the great difficulties and barriers encountered in Spain for the implementation of the Convention and, particularly, Article 19 [89], as also does the Committee on the Rights of Persons with Disabilities in its concluding observations on the combined second and third periodic reports of Spain [48], in which “the lack of a strategy for deinstitutionalization and of an action plan to promote independent living for all persons with disabilities in their community” (p. 9) is reported, as public funds continue to be invested in the construction of new residential institutions for this population group. A reform of the system is therefore urgently needed and should abandon mere assistance or rehabilitation approaches in order to prioritize funding of support systems (not necessarily linked to a specific and disability-related service) focused on the life project that the person, together with his or her circle of support, wishes to build.

This study reveals the need for the implementation and monitoring of strategies at the mesosystem level that may truly guarantee the right to living independently and participating in the community of people with extensive support needs. In this regard, it would be interesting for future lines of research to engage in a longitudinal assessment of the changes in the quality of life and especially in self-determination, of this population group, following processes of deinstitutionalization and transformation of the support model, including the perspective of professionals, relatives and people with ID themselves since experiences of individuals with extensive support needs are often overlooked [90].

## 5. Conclusions

This study has assessed the professional practices aimed at promoting the right of people with ID and extensive support needs to live and participate in their community, alongside other related rights, such as that to privacy or to habilitation and rehabilitation. The results obtained reveal that people with extensive support needs receive less support in terms of guaranteeing their right to independent living and privacy. Lastly, emphasis must be placed on the importance of performing well-planned deinstitutionalization processes that are not just limited to mere relocation in other spaces. Organizations that provide care for people with ID should transform their support model from a traditional approach to rehabilitation into one that is linked to the rights gathered in the Convention [1] and to the improvement of individual quality of life [84]. The use of valid and reliable indicators such as the ones presented, which enable monitoring of the appropriate implementation of measures, can be useful to progress towards full exercise of the rights of people with ID and, most especially, of those who have extensive support needs.

## Figures and Tables

**Table 1:** Sociodemographic characteristics.

Sociodemographic Variables	ESN * (n = 470)	LSN * (n = 259)	Total (N = 729)
**Age**			
Range	18–76	20–78	18–78
M (SD)	37.05 (12.79)	39.06 (12.57)	37.76 (12.74)
**Sex**			
Male %(n)	57.4 (270)	54.8 (142)	56.5 (412)
Female %(n)	42.6 (200)	45.2 (117)	43.5 (317)
**Diagnosis**			
ID %(n)	51.3 (241)	76.1 (197)	60.1 (438)
Down Syndrome %(n)	14.7 (69)	11.2 (29)	13.4 (98)
Cerebral Palsy %(n)	16.4 (77)	4.2 (11)	12.1 (88)
ASD %(n)	11.1 (52)	6.9 (18)	9.6 (70)
Other %(n)	6.6 (31)	1.5 (4)	4.8 (35)
**Severity of ID**			
Mild %(n)	6.8 (32)	37.5 (97)	17.7(129)
Moderate %(n)	23.0 (108)	51.4 (133)	33.1 (241)
Severe %(n)	50.0 (235)	9.3 (24)	35.5 (259)
Profound %(n)	18.9 (89)	0.4 (1)	12.3 (90)
Unknown %(n)	1.3 (6)	1.6. (4)	1.9 (10)
**Other disabilities** %(n)	63.0 (296)	41.7 (108)	55.4 (404)
**Residential Setting**			
Family (receiving professional support in family home) %(n)	66.1 (310)	70.3 (182)	67.5 (492)
Residential facility or nursing home %(n)	31.5 (148)	20.8 (54)	27.7 (202)
Group Home %(n)	2.3 (11)	8.9(23)	4.7 (34)

* ESN = Extensive support needs; LSN = Low support needs.

**Table 2:** Items addressing each article.

Convention Article	Items
Article 19. Right to Living Independently and Being Included in the Community	Regularly participates in community activities The person with disability is who ultimately and with the relevant support makes choices and decisions related to aspects of daily living (what clothes to wear, who they interact with, the activities they perform…) The person with disability has chosen the people who are part of his/her personal support circle The organization provides the necessary support so that, if desired, the person may live in his/her home, regardless of age or disability
Article 22. Respect for Privacy	The person has decided who may access his/her personal information The person has a private and intimate space inside the place where he/she lives The person’s privacy and intimacy are respected in the services provided by the organization (e.g., knocking before entering, closing the door when showering the person, when the person is using the toilet, etc.)
Article 26. Habilitation and Rehabilitation	The person’s interests are closely observed to provide activities that may respond to the person’s preferences The aim of the support provided by the organization’s professionals is that the person may achieve growing levels of participation in daily activities The approach of the individual support plan is multidimensional and holistic to improve the person’s quality of life The individual support plan includes clear strategies and enough supports to enable the person’s inclusion in ordinary settings

**Table 3:** Parameters of the ESEM (exploratory structural equation modeling) final model.

Item/Factor	F1	F2	F3	iECV
A1	**0.47**	−0.04	0.28	0.73
A2	**0.60**	0.15	0.07	0.93
A3	**0.94**	−0.01	−0.04	0.99
A4	**0.48**	0.28	0.00	0.75
B1	0.26	**0.44**	−0.02	0.74
B2	0.01	**0.74**	0.05	0.99
B3	0.02	**0.68**	0.10	0.98
C1	−0.08	0.06	**0.89**	0.99
C2	0.11	−0.02	**0.85**	0.98
C3	−0.11	0.05	**0.84**	0.98
C4	0.14	−0.06	**0.75**	0.96
ω	0.70	0.66	0.90	

Notes: iECV = item explained common variance. In bold = primary/targeted loadings. A, B and C factors correspond to professional practices oriented to promote the rights included in Articles 19, 22, and 26 of the Convention, respectively.

**Table 4:** MIMIC (multiple indicators-multiple causes) models.

Covariable	Model	df	CS	RMSEA	CFI	TLI
Sex	Saturated	24	70	0.055	0.992	0.979
	Invariant	32	71	0.044	0.994	0.987
	Null	**35**	**53**	**0.029**	**0.997**	**0.994**
Age	Saturated	24	67	0.056	0.993	0.980
	Invariant	32	65	0.042	0.994	0.988
	Null	**35**	**54**	**0.031**	**0.997**	**0.994**
Support needs	Saturated	24	71	0.055	0.993	0.979
	Invariant	32	84	0.051	0.992	0.983
	Partial invariant	**31**	**72**	**0.046**	**0.993**	**0.986**
	Null	35	126	0.064	0.986	0.973
Disability	Saturated	24	65	0.053	0.993	0.981
	Invariant	32	75	0.047	0.993	0.985
	Null	**35**	**66**	**0.038**	**0.995**	**0.990**
Comorbidity	Saturated	24	72	0.056	0.992	0.979
	Invariant	32	77	0.047	0.993	0.985
	Null	**35**	**59**	**0.033**	**0.996**	**0.993**
Home	Saturated	24	72	0.056	0.992	0.975
	Invariant	**40**	**81**	**0.040**	**0.993**	**0.987**
	Null	46	139	0.056	0.985	0.975

Note: df = degrees of freedom; CS = chi-square; RMSEA = Root mean error of approximation; CFI = Comparative fit index; TLI = Tucker-Lewis index; in bold = retained model.

**Table 5:** Fit of final SEM models.

Model	df	CS	RMSEA	CFI	TLI
Exhaustive	79	108	0.026	0.994	0.991
Parsimonious	74	128	0.027	0.995	0.992
Interaction	**107**	**131**	**0.020**	**0.996**	**0.994**

Note: df = degrees of freedom; CS = chi-square; RMSEA = Root mean error of approximation; CFI = Comparative fit index; TLI = Tucker-Lewis index; in bold = retained model.

## Data Availability

The data presented in this study are available on request from the corresponding author.
